# Determinants of weight outcomes in type 2 diabetes prevention intervention in primary health care setting (the DE-PLAN project)

**DOI:** 10.1186/s12889-017-4977-1

**Published:** 2018-01-02

**Authors:** Aleksandra Gilis-Januszewska, Beata Piwońska-Solska, Jaana Lindström, Ewa Wójtowicz, Jaakko Tuomilehto, Peter E. H. Schwarz, Katarzyna Kissimova-Skarbek, Zbigniew Szybiński, Adam Windak, Alicja Hubalewska-Dydejczyk

**Affiliations:** 1 Department of Endocrinology, Jagiellonian University, Medical College, ul. Kopernika 17, 31-501 Krakow, Poland; 2 Chronic Disease Prevention Unit, National Institute for Health and Welfare(THL), Helsinki, Finland; 3 Centre for Vascular Prevention, Danube-University Krems, Krems, Austria; 4 Department of Chronic Disease Prevention, National Institute for Health and Welfare, Helsinki, Finland; 5 Diabetes Research Group, King Abdulaziz University, Jeddah, Saudi Arabia; 6 Dasman Diabetes Institute, Dasman, Kuwait; 7 Department for Prevention & Care of Diabetes, Medical Clinic Unit III, University Clinic Carl Gustav Carus at Technical University Dresden, Dresden, Germany; 8 Department of Health Economics and Social Security, Institute of Public Health, Jagiellonian University, Medical College, Krakow, Poland; 9 Department of Family Medicine, Chair of Medicine and Gerontology, Jagiellonian University Medical College, Krakow, Poland

**Keywords:** Type 2 diabetes, Prevention, Lifestyle intervention, Diet, Physical activity, Weight loss, Determinants of weight loss, Real life setting

## Abstract

**Background:**

Real life implementation studies performed in different settings have proved that lifestyle interventions in the prevention of type 2 diabetes (DM2) can be effective, although the weight reduction results are typically modest compared to randomized control trials. Our objective was to identify the factors that predict successful weight loss in a less intensive, lower budget, real life setting lifestyle diabetes prevention intervention.

**Methods:**

Study participants (*n* = 175) with increased DM2 risk (Finnish Diabetes Risk Score (FINDRISC) > 14) but no diabetes at baseline received ten group lifestyle counselling sessions, physical activity and motivation sessions during a ten-month intervention. Stepwise regression analysis was used to determine demographic, clinical, and lifestyle predictors of successful weight reduction defined as a reduction of ≥5% of the initial body weight.

**Results:**

At 12 months following the initiation of the intervention, 23.4% of study participants lost ≥5% weight (mean loss of 7.9 kg, SD = 5.8). Increased physical activity (44% vs 25%, *p* = 0.03), decreased total fat consumption (88% vs 65%, *p* = 0.006) and adherence to four-five lifestyle goals (71% vs 46%, *p* = 0.007) were more often reported among those who managed to lose ≥5% weight versus those who did not.

In a multivariate analysis, meeting the ≥5% weight loss goal was most effective in individuals with a higher baseline BMI (OR 1.1, 95%CI 1.0–1.2), baseline and medium versus higher education (OR 5.4, 95% CI 1.2–24.7) and a history of increased glucose (OR 2.6, 95%CI 1.1–1.3). A reduction of total fat in the diet was an independent lifestyle predictor, increasing the probability of successful weight loss by 3.8 times (OR 3.8, 95% CI 1.2–11.4).

**Conclusion:**

Baseline higher BMI, lower education and a history of increased glucose predicted the successful weight loss among individuals with a high risk for the DM2 following lifestyle intervention in a real life primary health care setting. People who manage to lose weight more often adhere to lifestyle changes, while the reduction of total fat in diet independently predicts successful weight loss. Further studies exploring the predictors of success in implementation studies in DM2 prevention should help health care providers redesign interventions to improve their effectiveness and outcomes.

**Trial registration:**

ISRCTN, ID ISRCTN96692060, registered 03.08.2016 retrospectively.

## Background

Lifestyle intervention, through dietary and physical change, is very effective in type 2 diabetes prevention and as demonstrated in several studies can reduce DM2 incidence up to 60% [1–4]. Weight loss was the predominant predictor of DM2 prevention with 16% diabetes risk reduction for every kilogram of weight reduction [5]. Real life implementation studies performed in different settings and populations have also proved that less-intensive, lower budget lifestyle interventions can be effective, also for the long term [6–18].

The mean weight loss and the number of people who lost ≥5% of their initial body weight is much higher in randomized control studies (RCTs) than in less intensive and less costly real life implementation studies. Weight loss in RCTs has been shown to be greater for those who were older, had a higher diabetes risk, were engaged in more frequent self monitoring of fat intake, reported lower percentage calories from fat, increased consumption of fiber, and increased physical activity [5, 19–24]. Important psychological and behavioral predictors of weight outcomes have also been identified [24–27]. However, very little is known about the predictors of success in real life diabetes prevention intervention studies. Therefore, the purpose of this study was to examine the determinants of successful weight reduction among high DM2 risk participants during a lifestyle intervention in a primary health care setting.

## Methods

The DE-PLAN project (Diabetes in Europe: Prevention using Lifestyle, physical Activity and Nutritional intervention), EU initiated and sponsored, based on the principles of the Diabetes Prevention Study [1] was developed as a real life implementation study in 17 countries in Europe [28]. As the efficacy of lifestyle interventions have been well established by earlier diabetes prevention trials and given that the purpose of the DE-PLAN project was to examine the implementation of the intervention in real life settings, the need for additional randomized controlled trial study design in the current program was considered unnecessary and unethical.

A detailed description of the program including the inclusion criteria, the characteristics of the participants, methods, the intervention, and one-year and follow up results have been published previously [6, 7, 28].

### Study population

The study was performed in nine independent Primary Health Care General Practitioners’ (GP) practices in Krakow, Poland. The study group consisted of everyday patients, city inhabitants, aged over 25. The inclusion criterion was a high DM2 risk assessed with the Finnish Diabetes Risk Score (FINDRISC > 14) (33% chance of developing diabetes within 10 years). The exclusion criteria was either known diabetes or oral glucose tolerance test (OGTT) screened diabetes as well as known chronic disease which could affect the results of the study.

Information about the study and leaflets with the FINDRISC questionnaire were distributed in co-operating practices. Patients with known risk factors were directly approached by nurses and medical staff. Out of 800 FINDRISC questionnaires distributed, 566 were completed and 368 respondents scored FINDRISC > 14. Subsequently, 275 people signed informed consent and agreed to undergo OGTT examination. Of these 262 (258 with all measurements done) were invited to participate in the intervention. 184 participants completed the intervention (the number of completed sessions among completers was from eight to eleven), nine participants completed all sessions but not the final examination after one year and were excluded from the final analyses.

175 participants with complete baseline and one year data were included in the analyses.

### Description of intervention

The intervention followed the steps of the Diabetes Prevention Study (DPS) modified and adjusted to a local primary health care setting [1, 6, 7, 20, 28]. Well-trained nurses (two per center), certified in diabetes prevention, delivered a ten month intervention based on reinforced behaviour modification focusing on weight loss, reduced intake of total fat, reduced intake of saturated fat, change of saturated to unsaturated fat, increased consumption of fibre (from fruits, vegetables and cereal) and an increase of physical activity. The weight loss aim was to loss ≥5% of the initial body weight [1, 6, 7, 20, 28].

The initial intensive phase of the intervention (four months) consisted of one individual session followed by ten group sessions (10–14 people), focusing on diet and physical activity changes. During each session, printed educational materials related to the topic of the session were distributed. Social support was emphasized by the group setting and participants were also encouraged to invite their own social environment to the lifestyle changes. A spouse or other family member could also participate in the sessions. From week four of the initiation of the intervention, patients were offered physical activity sessions (aqua aerobics and gymnastics or football) twice weekly and free of charge. The ongoing maintenance phase of the intervention (month 4–10) following the intensive phase consisted of six motivational telephone calls and two motivational letters [1, 6, 7, 20, 28].

There was no other post-intervention contact with the participants except for measurements taken at one year.

This study followed the Good Clinical Practice guidelines and the guidelines of the Helsinki Declaration. All study participants gave their written informed consent prior to the participation in the study.

### Measurements, predictors and outcome variables

Patients were examined at baseline and after 12 months of the study. The examination procedure included: standardized questionnaires (FINDRISC, baseline, clinical, lifestyle) and biochemical tests including: fasting and 120’OGTT glucose, serum triglycerides, HDL and total cholesterol. Impaired Fasting Glucose (IFG) was defined as a fasting plasma glucose concentration of 6.1 to 7.0 mmol/l. Impaired Glucose Tolerance (IGT) was defined as a glucose plasma concentration of 7.80 to 11.0 mmol/l after oral administration of 75 g of glucose (OGTT). Diabetes mellitus (DM) was defined as fasting glucose concentration of more than 7.0 mmol/l or a glucose concentration of more than 11.1 mmol/l at two hours of OGTT [6, 7]. Body mass index (BMI) was calculated as weight (kg) divided by height squared (m^2^). Waist circumference was measured midway between the lowest rib and the iliac crest. Diastolic and systolic blood pressures were taken while sitting following 10 min rest.

Data regarding education, marital status, employment status, history of increased blood glucose, family history of diabetes, diabetes risk score FINDRISC, smoking status, history of hypertension, history of cardiovascular disease (CVD) and history of depression were taken with the use of self-reported questionnaires.

Lifestyle changes were also explored with the use of self reported standardized questionnaires regarding the: increase of consumption of vegetables and fruits, decrease of consumption of total fat, decrease consumption of saturated fat, change of saturated fat to unsaturated, decrease of alcohol consumption and increase of physical activity over the past year.

Lifestyle goals’ achievement was defined as low if one-three goals were achieved and high if four-five goals were achieved.

Participants were categorized into two groups based on the weight reduction achieved at 12 months using a ≥ 5% weight reduction as the cut-off point.

### Statistical analyses

Chi-square test for categorical variables and t-test for continuous ones were applied to compare the distribution of the potential predictors in groups of the participants who lost ≥5% of initial body weight compared to those who did not. Stepwise logistic regression models were used to assess the association between the different predictors and the outcome variable. The odds ratios and the respective 95% confidence interval were calculated. Coefficients of contingency were calculated to assess correlations between the lifestyle variables separately for those who achieved loss of ≥5% of initial body weight and those who did not (max. C for tables 2 × 2 = 0.707).

The data was analyzed using STATISTICA 12. A *p*-value of <0.05 was considered statistically significant.

## Results

175 participants (22% men, mean age 56.1 (SD = 10.9), mean BMI 31.8 (SD = 5.0) completed the core curriculum and participated in the final examination. Men were younger, had a higher BMI and waist circumference, higher serum TG and lower HDL, and had more often IFG than women (*p* < 0.05) (data not shown). Of the participants, 69% were married or had a partner, 21% were current smokers. Family history of diabetes, history of increased glucose, CVD history, hypertension history and depression history was present in 60%, 60%, 37%, 66% and 16% of participants respectively.

Of the participants, 21% had a higher education and 39% were working. Among the participants with a higher education, 61% were working, while among those with a baseline/medium education, 34% were working (*p* = 0.004).

At 12 months after the initiation of the intervention, the participants’ mean weight was reduced by 1.9 ± 5.0 kg (2.1%) and 23.4% lost ≥5% of their initial body weight (mean weight reduced by 7.9 kg SD = 5.8); 88% of those who managed to loose ≥5% of weight were women.

People who succeeded to lose weight had a higher BMI at baseline (33.3, SD = 4.7 vs 31.3, SD = 5.0, *p* = 0.02) and less often had a higher education (5% vs 25% p = 0.004) (Table 1). There were no other baseline demographic, anthropometric or lifestyle differences between those who managed or did not manage to lose ≥5% of their weight.

**Table 1:** Baseline characteristic of participants in groups of body weight change: body weight reduction <5%, body weight reduction ≥ 5%

	<5% body weight reduction (*n* = 134)		≥5% body weight reduction (*n* = 41)		
	mean/%	SD	Mean/%	SD	*P*
Age	55.6	11.5	57.8	8.9	0.256
% men	25		12		0.066
Weight (kg)	85.0	16.6	87.8	14.5	0.321
BMI (kg/m2)	31.3	5.0	33.3	4.7	0.020
WC (cm)	98.0	12.1	101.2	10.5	0.133
SBP (mmHg)	132.2	14.5	132.1	14.3	0.964
DBP(mmHg)	82.2	8.8	81.5	7.8	0.667
Fasting glucose (mmol/l)	5.3	0.7	5.3	0.8	0.691
2-h OGTT glucose (mmol/l)	6.0	1.8	5.5	1.8	0.190
TCH(mmol/l)	5.6	1.0	5.4	0.9	0.137
HDL(mmol/l)	1.4	0.4	1.4	0.3	0.687
TG(mmol/l)	1.8	1.2	1.7	1.0	0.799
FINDRISC	18.2	2.8	18.8	3.0	0.203
NGT%	71		83		0.219
IFG %	11		5.0		0.367
IGT%	16		12		0.626
Education basic and medium %	75		95		0.004
Education high %	25		5		
Married/having a partner	69		71		0.849
Single /widow	31		29		
Working	42		32		0.511
Retired	52		61		
Not working	6		7		
Smoking currently	23		12		0.184
History of Increased Glucose	57		71		0.145
History of Hypertension	66		68		0.851
History of Hyperlipidaemia	53		51		0.86
History of Depression	12		17		0.311
History of CVD	43		34		0.176
Family history of DM2	62		51		0.206

Key: *BMI* body mass index, *SBP* systolic blood pressure, *DBP* diastolic blood pressure, *OGTT* oral glucose tolerance test, *TCH* total cholesterol, *HDL* high density lipoprotein, *TG* triglicerides, *IFG* impaired fating glucose, *IGT* impaired glucose tolerance, *NGT* normal glucose tolerance, *History of CVD* history of cardiovascular disease

**Fig. 1:**
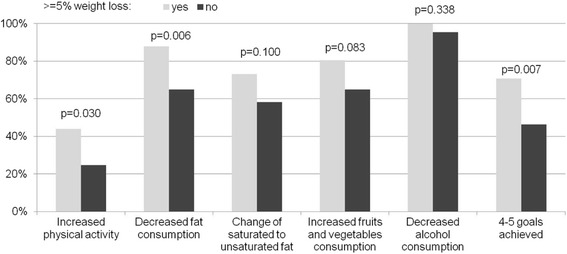
Lifestyle changes and lifestyle goals achieved after intervention in groups of study participants who lost or did not lose ≥5% body weight

An increase in physical activity (44% vs. 25%, *p* = 0.03) and decrease in fat consumption (88% vs. 65%, *p* = 0.006) was more often reported among those who achieved ≥5% weight reduction (Figure 1). Participants who managed to lose ≥5% of their weight more often adhered to 4–5 lifestyle goals than those who did not (71% vs 46%, *p* = 0.007).

In the analyses of contingency between lifestyle changes in people who did not manage to lose ≥5% weight, there was a weak correlation between the increase in physical activity and dietary changes, while the only strong correlation was observed between decrease of total fat consumption and decrease of saturated fats C = 0.46 and decrease of total fat in diet and increase of consumption of vegetables and fruit C = 0.43 (max. C for tables 2 × 2 = 0.707).

In participants who managed to lose ≥5% of their weight, an increase of physical activity was correlated with the decrease of saturated fats consumption C = 0.39, decrease of total fat consumption C = 0.31 and increase of fruits and vegetable consumption C = 0.30. The decrease of total fat consumption was strongly correlated with the decrease of saturated fats C = 0.52 and increase of vegetable and fruits consumption C = 0.49. The decrease of saturated fat consumption was also correlated with an increased consumption of vegetables and fruits C = 0.37 (Table 2).

**Table 2:** Analyses of contingency between lifestyle changes in people who did or did not manage to lose ≥5% of weight (max. C for tables 2 × 2 = 0.707)

	Decreased consumption of fat over past year	Changed the saturated fat to unsaturated fat used during the past year	Increased consumption of fruits and vegetables over past year
People who managed to lose ≥5% of weight			
Increased physical activity over past year	.31	.39	.30
Decreased consumption of fat over past year		.52	.49
Changed the saturated fat to unsaturated fat used during the past year			.37
People who didn’t manage to lose ≥ 5% of weight			
Increased physical activity over past year	.20	.23	.20
Decreased consumption of fat over past year		.46	.43
Changed the saturated fat to unsaturated fat used during the past year			.34

In multivariate analysis, meeting the ≥5% weight loss goal was most effective in individuals with baseline higher BMI (OR = 1.1, 95% CI 1.0–1.2), baseline and medium education (OR = 5.4, 95% CI 1.2–24.7) and history of increased glucose (OR = 2.6, 95% CI 1.1–1.3). Among lifestyle changes, reduction of total fat in diet was an independent predictor, increasing the probability of successful weight loss by 3.8 times (OR = 3.8, 95% CI 1.2;11.4). No other baseline and lifestyle factors modified the effect of weight loss (Table 3).

**Table 3:** Multivariate analysis of predictors of ≥5% weight loss after one year of intervention

	Basic model		Final model	
	OR	95% CI	OR	95% CI
Age	1.0	0.9–1.1		
Sex M vs F	0.4	0.1–1.7		
Education baseline and medium vs high	7.4	1.4–38.0 *p* = 0.016	5.4	1.2–24.7 *p* = 0.029
Marital status married or having a partner vs single or widow	0.8	0.3–2.0		
Working vs not working retired	0.6	0.2–2.0		
BMI	1.1	1.0–1.3	1.1	1.0–1.2 *p* = 0.006
Waist circumference	1.0	0.9–1.1		
FINDRISC	0.9	0.8–1.1		
History of Increased Glucose	3.5	1.1–10.9 *p* = 0.031	2.6	1.1–6.1 *p* = 0.028
Family History of Diabetes	0.5	0.2–1.6		
History of CVD	1.3	0.5–3.3		
History of Hypertension	0.8	0.3–2.4		
Smoking currently	0.4	0.1–1.2		
Increased physical activity over past year	2.0	0.8–5.2	1.9	0.9–4.4 p = 0.109
Decreased consumption of total fat over past year	3.3	0.8–13.7	3.8	1.2–11.4 *p* = 0.019
Increased consumption of fruits and vegetables over past year	1.1	0.3–3.4		
Change of saturated to unsaturated fat over past year	1.4	0.5–4.2		

Key: *BMI* body mass index, *History of CVD* History of Cardiovascular Disease

## Discussion

This is one of the first studies examining the predictors of weight modification outcomes in a real life, real setting diabetes prevention intervention, among high diabetes risk participants. We have reported earlier that DM2 prevention through lifestyle intervention in a primary health care setting is feasible and effective with results of modest weight reduction with ≥5% weight loss in 23.4% of study participants [6]. We have also recently reported that weight loss, however modest, with beneficial metabolic outcomes can be maintained at a 3-year follow-up [7].

In the current analyses we found that successful weight loss was independently predicted by a higher baseline BMI, lower education and a history of higher glucose, while among lifestyle factors, the reduction of total fat in the diet was a strong, independent predictor of successful weight reduction. An increase in physical activity, decrease of total fat consumption and better adherence to lifestyle goals was more often reported among those who managed to lose weight versus those who did not. In the participants who managed to successfully lose weight, an increase of physical activity was correlated with beneficial dietary changes, while in people who did not manage, this correlation was very weak.

The evidence from RCTs confirm that weight loss is the predominant predictor of diabetes prevention: in the Diabetes Prevention Program (DPP) 55% of the reduction in incidence of DM2 over 3 years follow- up was explained by a loss of 5 kg [5]. Diabetes prevention in people at high risk is one of the most important challenges in primary health-care, although the results of the translation studies are modest [6–18]. Therefore, the improvement of the efficacy in implementation programs is one of the biggest challenges to the public health sector. Thus, there is a need to identify predictors and barriers of weight loss in DM2 prevention programs in primary health-care patients.

It is important to remember that interventions given in RCTs are not easily replicated in translational studies, where lower resources lead to less intensive intervention. Interventions also need to be adapted to local, cultural and health care possibilities. As a consequence, as seen also in our study, the achieved weight reduction usually is lower compared with RCTs [6–10, 12–14, 17, 18]. Also, the percentage of people who lost ≥5% of their initial weight was substantially lower in our study than in the DPS or DPP (>7% of body weight reduction) studies (23.4% vs 37% and 37% respectively) [1, 2, 4, 5].

In previous RCTs, weight loss was greater for those who were older [4, 5, 19, 20], had higher diabetes risk [20], were engaged in more frequent self monitoring of fat intake, reported lower percentage calories from fat, increased consumption of fiber and increased physical activity [4, 5, 19–23].

In our study, a higher baseline BMI, which is a marker of increased diabetes risk, predicted the successful loss of weight. This finding is concordant with results of a study by Wadden et al. where the most consistent predictor of absolute weight loss was initial body weight, with heavier individuals losing more weight [29].

In our study older age was not related to weight loss success. This is in contrast to the DPP and DPS results, where older age was a strong predictor of success at meeting the weight loss goal [19, 20]. Our results were, however, in concordance with other publications [30] where the reason of no association with age could be explained with the small age ranges of the studied populations.

Gender was also not related to weight outcomes, as seen in other studies [4, 5, 19–22]. However our study, like some others, attracted mainly woman. Only 22% of the participants were men, while among those who managed to lose ≥5% weight only 12% were men. This highlights the well- known need to develop lifestyle interventions further to increase male participation and to focus more on how to address interventions to improve the success rate in men [19, 20].

The relation between lower education (baseline/medium vs high) and better weight outcomes, however surprising, might be explained by the almost double higher percent of working people among those with a higher education. This is concordant with other studies showing that lifestyle intervention programs are taken up mostly by non-working people [31–33]. Schedule conflicts with working hours could be the main obstacle in the uptake of the diabetes and cardiovascular disease prevention services [31–33]. Therefore, to improve the reach, attendance and the outcomes of prevention initiatives among working people, several new strategies targeted towards providing accessible services are being investigated such as telephone and internet based interventions, mobile apps or workplace- run interventions [8, 34–40]. For example, in Finland, during the diabetes prevention program among airline employees the uptake of the group intervention was so low that this intervention was discontinued; instead, a well accepted, diabetes prevention website was developed which in turn was very well received [8].

In our study, the history of increased glucose reported at baseline (original question was “Have you ever been told by any medical professional that you had increased glucose”) was also an independent predictor of successful weight loss. “Medical triggers” like information regarding already existing diabetes or CV risk have been previously reported as factors leading to successful weight loss [41]. suggesting that medical advice and awareness of the risk of disease are important to achieve weight loss. In our study, summarized information regarding history of increased glucose, family history of diabetes, as well as a summary of anthropometric and biochemical results were presented during the first individual session to explain the individual diabetes risk as one of the motivation tools.

Other socioeconomic variables including marital and smoking status were not related to weight loss success which is consistent with some other publications [19, 20, 23]. In some studies depression with coexisting symptoms like lack of motivation, uncontrolled eating and alcohol abuse have been inversely related with weight loss success [42]. In our study, the proportion of participants with depression was very low and therefore it is difficult to draw any conclusion on such an association.

In the DPS the intervention was found to be most effective among those with a high baseline diabetes risk assessed by the FINDRISC. The Number Needed to Treat (NNT) was 7.7 and 3.6 for the low and high FINDRISC, respectively [20]. In the DEPLAN study we decided to use the simple questionnaire for inclusion and the criterion (FINDRISC > 14) was proposed based on the results of the DPS, suggesting that selecting people with high diabetes risk assessed with this easy questionnaire helps to find people who benefit most from the intervention. No association between successful weight loss and baseline FINDRISC in our study could be explained with the high inclusion value and therefore small range of FINDRISC score.

In our study, among the investigated changes of lifestyle factors, the decrease in dietary fat consumption was a strong independent factor of successful weight loss. In both groups of weight loss we also observed favorable increase of vegetable and fruit consumption and change of saturated to unsaturated fats however the differences between the groups were not significant. In analysis of contingency in both groups there was a correlation between the total fat consumption decrease and change of saturated fats to unsaturated and increase consumption of vegetables and fruit. In the DPS, individuals with low fat and high fiber intake lost more weight than those consuming a high fat, low fiber diet [21]. Similarly in the DPP, a lower percent of calories from fat and increased physical activity were predictors of weight loss; for every 5% reduction in percent fat in diet during follow-up, the diabetes incidence was reduced by 25% [5]. Among the participants who did not meet the weight loss goal, there was no significant effect of meeting the percent fat goal on diabetes incidence [5]. In our study, the increase of physical activity also increased the probability of successful weight loss by almost two times. Although this association was not significant in the stepwise analysis model (*p* = 0.109) and the decrease of fat consumption was the strongest lifestyle predictor of success, increased activity was not meaningless (95% CI 0.9–4.4). The lack of significance in the stepwise analyses model might be explained by the correlation between physical activity increase and decrease of total fat consumption. In the DPS, individuals who increased moderate to vigorous and strenuous, structured leisure time physical activity resulted in 63–65% reductions in diabetes risk, even after adjustment for changes in weight [22]. Also in the DPS it was shown that risk reduction was dependent on adherence to the lifestyle intervention goals [1, 4]. Similarly in our study, people who managed to achieve ≥5% weight loss vs those who did not more often managed to achieve 4–5 lifestyle goals. As much as 75% of people who did not achieve weight loss did not increase physical activity, while 35% did not decrease total fat in diet. In the participants who successfully lost weight, 55% did not increase physical activity and only 12% did not decrease total fat in their diet. In this group there was a correlation between the increase of physical activity and beneficial dietary changes, such as the decrease of total fat in diet, change of saturated to unsaturated fat, and an increase of the consumption of vegetables and fruit. In our study the adherence to physical activity seemed to be much more difficult than adherence to dietary changes, while an increase of physical activity in those who managed to lose weight was related to positive dietary changes. Therefore, while designing future prevention initiatives it seems that more emphasis should be placed on the methods to increase physical activity.

Some strength and limitations of our study need to be discussed. This is one of the first studies investigating the predictors of success in the real life, real setting implementation of type 2 diabetes prevention intervention. The participants in our study were volunteers, and like seen in many other studies, as discussed above, the study predominantly attracted women, with only 22% of the total cohort being men and only 12% of men among those who successfully lost weight. Therefore, our results may not be generalizable to both sexes. In addition, the modest weight reduction obtained in our study might be influenced by the female sex domination, whose success in previous diabetes prevention studies was meager when compared to men [43].

In light of the very poor male participation and success, these factors should also be further investigated, separately for both sexes. The sample size was relatively low and may have caused issues in power to detect small effects. The changes in lifestyle were assessed with the use of self- reported questionnaires also used in the DPS, while in some of the RCTs other methods assessing physical activity and diet were used. To deepen our analysis regarding lifestyle changes, we also investigated the correlations between physical activity and dietary patterns. What needs to be added is that in our study we have not examined psychological and behavioural factors influencing weight outcomes [23–27, 30, 42].

Further studies exploring predictors of success in implementation studies in DM2 prevention should help health care providers redesign interventions with special focus being placed on physical activity to improve their effectiveness and outcomes.

## Conclusion

Additional insight into participant characteristics, including psychological and behavioral factors that independently predict weight loss, is critical for the further efforts of health care providers in real life diabetes prevention initiatives to identify those who are most likely to succeed and to understand the barriers of those who are not successful.

## Abbreviations

CVD: Cardiovascular disease
DA QUING: the China Da Qing Diabetes Prevention Study
DE-PLAN: Diabetes in Europe: prevention using lifestyle, physical activity and nutritional intervention
DM 2: Diabetes mellitus type 2
DPP: Diabetes prevention program
DPS: Diabetes prevention study
IFG: Impaired fasting glucose
IGT: Impaired glucose tolerance
NGT: Normal glucose tolerance
OGTT: Oral glucose tolerance test
RCT: Randomised control studies
